# Assessing the sociability of former pet and entertainment chimpanzees by using multiplex networks

**DOI:** 10.1038/s41598-020-77950-x

**Published:** 2020-12-01

**Authors:** Dietmar Crailsheim, Toni Romani, Miquel Llorente, Elfriede Kalcher-Sommersguter

**Affiliations:** 1 Unitat de Recerca i Etologia, Fundació MONA, Riudellots de La Selva, Spain; 2 Facultat d’Educació i Psicologia, Universitat de Girona, Girona, Spain; 3 Faculty of Artes Liberales, University of Warsaw, Warsaw, Poland; 4 Institut de Recerca i Estudis en Primatologia - IPRIM, Girona, Spain; 5 Institute of Biology, University of Graz, Graz, Austria

**Keywords:** Animal behaviour, Social behaviour

## Abstract

Advances in the field of social network analysis facilitate the creation of multiplex networks where several interaction types can be analysed simultaneously. In order to test the potential benefits of this approach, we investigated the sociability of atypically raised chimpanzees by constructing and analysing 4-layered multiplex networks of two groups of former pet and entertainment chimpanzees (*Pan troglodytes*). These networks are based on four social interaction types (stationary vicinity, affiliative behaviour, allogrooming, passive close proximity) representing low- to high-level interaction types in terms of sociability. Using the tools provided by the MuxViz software, we could assess and compare the similarity and information gain of each these social interaction types. We found some social interaction types to be more similar than other ones. However, each social interaction type imparted different information. We also tested for a possible impact of the chimpanzees’ biographical background on the social interaction types and found affiliative behaviour as well as allogrooming to be affected by adverse early life experiences. We conclude that this multiplex approach provides a more realistic framework giving detailed insight into the sociability of these chimpanzees and can function as a tool to support captive care management decisions.

## Introduction

Network approaches based on social behaviours of nonhuman animals facilitated the successful evaluation of how sociality is shaped by evolutionary and ecological conditions and reflected in behavioural processes, such as social learning^1^ and cooperation^2^, and also in the spread of diseases^3,4^. Furthermore, it enabled researchers to investigate, simulate and predict patterns of hierarchies^5^, information transmission efficiency^6^, group cohesion and stability^7,8^. Traditionally social networks were analysed by aggregating information and/or investigating only one type (e.g. a certain behaviour or distance) of connection between individuals. While this approach might seem narrow, it did allow the explanation of trends and patterns, which had been misinterpreted or underrated previously^9^. However, as research on social networks advanced over time and databases became bigger and more varied, the necessity arose to get insights in social networks that are even more realistic. Considering the multi-dimensional nature of the network components in space and time^10,11^, it became obvious that in order to fully grasp social structures and dynamics, it was essential to construct multiple social networks based on a variety of edges (i.e. connections between nodes) between the same set of nodes (i.e. individuals)^12^.

A great many studies on a variety of species, ranging from insects to nonhuman primates demonstrated the complexity of social structures in the animal kingdom^13,14^. Particularly for nonhuman primates, who are living in complex social societies, using a variety of strategies and behaviours to interact and connect with each other^15^, it seems a promising approach to implement a more realistic framework in order to explore their social structures. Especially during the last two decades, algorithms and computational technologies have been developed, providing the means to analyse and visualize complex multilayer relationships^16^. Hence, the use of these multilayer networks is now also recommended^17^ and it has already been used in the studies of primate behaviour^18,19^.

While it is recommendable to create a multilayer network based on several edges, the question of how many edges should be taken into account remains. Keeping in mind that an increase of data collected comes with a certain price, the right equilibrium between information gain, efficiency and redundancy has to be found^20,21^.

One of the most relevant social behaviours of chimpanzees is allogrooming^22,23^, which beside its hygienic function^24^ is used to establish and maintain relationships, bonds and coalitions^25^. As such, many studies investigating the social networks of chimpanzees focus on social grooming as their edge variable^26–30^. In cases where allogrooming is rare or difficult to observe, information might be limited to spatial or temporal co-occurrences of two individuals^31^.

Our latest long-term study on grooming networks in former pet and entertainment chimpanzees demonstrated variations in the grooming activity on an individual level based on the chimpanzees’ biographical background^30^. More precisely, we found wild-caught chimpanzees as well as chimpanzees who were predominantly housed without conspecifics during infancy to be more affected in their grooming activity and their distribution of grooming compared to those who were captive born as well as those who were predominantly housed with conspecifics during infancy. This could be explained by the fact, that similar to humans, the infancy in chimpanzees is a sensitive and crucial time period with respect to the social and emotional development^32,33^. Chimpanzee infants are heavily dependent on their mother and are nursed for their first five years of life. The loss of the mother causes behavioural disturbances and in case of unweaned infants may cause even the death of the infant^23,34–36^. With respect to the behavioural development of free-living chimpanzees, it is known that social play already occurs during the first month of an infant’s life^37^, whereas grooming starts to develop steadily at about the age of two years but is infrequent until the age of four years^38^.

Only recently, a study on wild living chimpanzees revealed the significance of maternal care on the survival of infant chimpanzees even beyond nutritional dependence^39^. Several studies demonstrated that atypical rearing conditions and traumatic experiences during this time period produce long-lasting negative effects in chimpanzees^40–42^, affecting among others, their social skills^43^, their personality profile^44^ and their cortisol levels at an adult age^45^. Bradshaw et al.^46^ and Ferdowsian et al.^47^ supposed that traumatic early life experiences in chimpanzees cause symptoms which they defined as Complex Post-Traumatic Stress Disorder (PTSD), and these symptoms are comparable to those of human trauma survivors who suffered highly distressing events at an early age. Some studies even documented atypical rearing conditions to lead to structural covariations of the gray matter in the brains of adult chimpanzees^48^.

Based on these findings we might expect not only allogrooming but also other social behaviours to be affected by the early life history of our study population. We were interested in how these former pet and entertainment chimpanzees with their atypical life histories would tolerate and deal with different types of social interaction. It has to be expected, that the experience of being caught in the wild and/or being housed without conspecifics during infancy, i.e. during their first five years of life, would affect the occurrence of certain interaction types, in particular those that require the toleration of permanent body contact and close proximity. However, these social interaction types might not be affected in the same way and/or to the same degree by the atypical life history, which would support the idea that analysing various potentially important social interaction types simultaneously might provide more precise and realistic results.

In previous studies^26,30^ we assessed chimpanzees sociability by looking into how their atypical life history affected their grooming activities based on their individual centrality scores. In this study, however, we chose a relational approach by taking the atypical life history not only of the individual but also of his/her partner into account when investigating their directed dyadic interactions.

To investigate this assumption, we chose four different social interaction types. Stationary vicinity (i.e. staying out of an arm’s reach but within 5 m without further interacting) represents a low-level interaction type in terms of sociability due to the distance between the individuals and as no bodily contact occurs. Affiliative behaviour (including behaviours such as social play and socio-sexual behaviours except for allogrooming) represents a medium-level interaction type due to a decrease in the distance of the interacting individuals and as bodily contact may occur. Allogrooming and passive close proximity represent high-level interaction types in terms of sociability as allogrooming requires the toleration of permanent body contact and passive close proximity (i.e. staying within an arm’s reach without further interacting) requires a certain amount of trust in the individual close by as the intention of that individual is, contrary to allogrooming, not immediately apparent. We used these different interaction types as they have already been tested and approved in severely deprived former laboratory chimpanzees^49^.

In the current study, we attempt to implement the use of a multiplex network analysis because it allows us to consider the four interaction types simultaneously. We want to find out if (1) the multiplex approach indeed increases the information gain compared to traditional single-layer and aggregate network analyses (even in small sized groups of 7 individuals), (2) (dis-)similarities might be found between the four interaction types, and (3) if there are individual differences in the occurrence of certain social interaction types. Furthermore, we were interested to see if potential differences detected between individuals and/or groups could be partially explained not only by the individual chimpanzee’s early life history but also that of his/her group members.

For this end, we created multiplex networks of the two groups of former pet and entertainment chimpanzees housed at Fundació Mona, consisting of seven individuals per group. The four layers of our multilayer networks are based on the four different social interaction types (explained in detail above): stationary vicinity, affiliative behaviour (except for allogrooming), allogrooming, and passive close proximity. We will evaluate each layer separately, its aggregated and multiplex components, and compare the obtained insights by using the open-source MuxViz software^16^. We predict that each layer will provide different information, and by taking all of the four layers into account, we expect to achieve a more detailed and realistic representation of the sociability of these two groups. More specifically, based on earlier findings in ex-laboratory chimpanzees, we expect to find an interlayer correlation between allogrooming and passive close proximity as both are representing high-level interaction types in terms of sociability. We expect to find an interlayer correlation between affiliative behaviour and allogrooming, as both interaction types require the toleration of body contact. We do not expect to find an interlayer correlation between stationary vicinity, representing a low-level interaction type, and either allogrooming or passive close proximity, representing high-level interaction types. Based on findings of long-term observations on this study population over a period of 12 years^30^ and on ex-laboratory chimpanzees^49,50^, we also expected the chimpanzees’ biographical background to have an effect on the four different social interaction types. Here, we predict allogrooming as well as passive close proximity to be affected by early life history, as the toleration of permanent body contact and the ability to perceive the group members as trust-worthy may be impaired in adversely reared chimpanzees. This should be reflected in a reduced (or even lacking) grooming activity as well as a reduced toleration of passive close proximity in individuals who were caught in the wild and/or predominantly housed without conspecifics during infancy compared to those individuals who were born in captivity and/or predominantly housed with conspecifics during infancy. We also expect affiliative behaviour to be affected by early life history, as it might be at least partly socially learned during infancy similar to allogrooming and thus might be reduced in individuals who are predominantly housed without conspecifics during infancy. We did not expect to find the toleration of stationary vicinity to be affected by the chimpanzees’ biography because in terms of sociability this is a low-level interaction type as the individuals are out of reach of each other. Beyond these four interaction types, we also tested the impact of the chimpanzees’ biographical background on the aggregated variable of these four interaction types, in order to check whether this aggregated variable produces an information loss as expected.

## Materials and methods

### Ethical note

This study is based purely on behavioural observations and was conducted in accordance with all national and institutional guidelines for the care and management of primates as established by Fundació MONA, the Association for the Study of Animal Behaviour/Animal Behavior Society and the Spanish Government (RD 53/2013).

### Study sample

The study sample consisted of a total of 14 former pet and entertainment chimpanzees (9 males and 5 females) living in two different social groups and housed at the primate rescue centre Fundació MONA in Catalonia, Northern Spain. The centre is a member of the European Alliance of Rescue Centres and Sanctuaries (EARS) and it is rehabilitating chimpanzees since 2001. Biographic information of the study subjects is presented in Table 1.

**Table 1:** Characteristics and background information on the study population.

Name	ID	Sex	Origin	Predominant housing condition during infancy (with or without conspecifics)	(Est.) year of birth	Year of arrival at MONA	Group
Bea	BEA	F	Wild-caught	With	1985	2012	Bilinga
Cheeta	CHE	F	Wild-caught	Without	1990	2015	
Coco	COC	F	Wild-caught	Without	1994	2012	
Nico	NIC	M	Captive born	Without	2001	2004	
Tico	TIC	M	Wild-caught	Without	1985	2005	
Tom	TOM	M	Wild-caught	With	1985	2011	
Victor	VIC	M	Captive born	Without	1982	2006	
Africa	AFR	F	Wild-caught	Without	2000	2009	Mutamba
Bongo	BON	M	Captive born	With	2000	2002	
Charly	CHA	M	Captive born	With	1989	2001	
Juanito	JUA	M	Captive born	With	2003	2005	
Marco	MAR	M	Captive born	With	1984	2001	
Toni	TON	M	Wild-caught	With	1983	2001	
Waty	WAT	F	Captive born	With	1996	2002	

_*F* female, *M* male_.

Both groups consisted of adult chimpanzees (Mutamba group: 5 males and 2 females, Bilinga group: 4 males and 3 females) and no changes to the group composition occurred during data collection for this study.

Observations were conducted only while the chimpanzees had access to one of the two enriched and naturalistic outdoor enclosures (size of 2 420 m^2^ and 3 220 m^2^, respectively) which gave them the opportunity to exploit natural and artificial resources. Group members of a social group could see but not physically interact with group members of the other social group. For more detailed information on the housing facilities see^51,52^.

The chimpanzees were fed four times per day with a balanced diet based on fruits, seeds and vegetables. They have limited quantities of other protein-rich foods (constant since 2001) and have access to water ad libitum. A big portion of their daily diet is scattered and hidden in the outdoor enclosures to stimulate natural foraging behaviour and locomotion as part of their daily enrichment program.

### Data sampling

Data on the chimpanzees’ behaviour and proximity were recorded between May 2018 and January 2019 by conducting two-minutes scan sampling^53,54^. One observation session lasted for 20 min where the behaviour, the proximity (passive close proximity), position and height within the enclosure of all the individuals of one group were recorded every two minutes simultaneously. Data was recorded between approximately 10.30 a.m. and 6.30 p.m., i.e. while the chimpanzees had access to the outdoor enclosure. The observation sessions were evenly distributed between mornings and afternoons on randomised days (Monday to Sunday). Observers were located in one of the two observation towers while conducting their observations, allowing them to oversee the respective enclosure. Observers (n = 9) were only allowed to collect data if they successfully passed a three-step inter observer reliability test. The first step included data collection over about two weeks; this data was checked and then deleted. In the second step observers have to pass a methodology test and in the third step they had to pass a video test that includes 20 different video clips with an agreement of ≥ 85 percent to the head of research.

Although a complete set of behaviours was recorded, for this study we only considered social interactions that occurred among group members and recorded if two individuals stayed within close proximity (i.e. within an arm’s reach). Furthermore, data on the chimpanzees’ position within the enclosure was recorded digitally on a GPS scaled enclosure map. Additionally, the observers recorded the height level of the chimpanzees (ground and four levels of the climbing structures, respectively). We calculated linear distance values between each pair of individuals of a group every two minutes, using the matrix distance plugin available in QGis 2.18^55^ and counted the pairs that were within 5 m (i.e. for the calculation of stationary vicinity) per scan. We corrected these values by substracting the occurrences where the respective pairs were within an arm’s reach, and also if the height level difference was more than one. Observers used tablets with the ZooMonitor data scoring software^56^ programmed with the sanctuary’s monitoring ethogram and facility map data. A total of 67 997 scans have been collected for this study (Bilinga group 32 320; Mutamba group 35 677).

### Data preparations

The edges represent the four social interaction types. Scan data was used to calculate index values of stationary vicinity (i.e. staying out of an arm’s reach but within 5 m without further interacting), affiliative behaviour (except for allogrooming), allogrooming and passive close proximity (i.e. staying within an arm’s reach without further interacting). The four indices are mutually exclusive (see Table 2 for edge definitions), i.e. if individual A is grooming individual B, these two individuals cannot be in close proximity simultaneously. Note, however, that individual A and B can be in close proximity or stationary vicinity to their other group members simultaneously.

**Table 2:** Definition of edge variables (i.e. indices).

	Edge	Definition	Calculation of index values
Social interaction types	Stationary vicinity	Being out of an arm’s reach but within a 5 m distance without further interacting	Number of scans where individual A and individual B where out of an arm’s reach but within a 5 m distance divided by the number of scans where individual A and individual B had access to each other
	Affiliative behaviour	Including social play, socio-sexual and other affiliative behaviours such as follow^a^, embrace, feed together, touch, mouth-to-mouth, short body contact, extend arm (except for allogrooming)	Sum of the number of scans where individual A exhibits affiliative behaviour towards individual B divided by the number of scans where individual A and individual B had access to each other
	Allogrooming	Cleaning and/or manipulating the hair/body of a group member (unidirectional or mutual)	Sum of the number of scans where individual A is grooming individual B divided by the number of scans where individual A and individual B had access to each other
	Passive close proximity	Being within an arm’s reach without further interacting	Number of scans where individual A and individual B were within an arm’s reach divided by the number of scans where individual A and individual B had access to each other

All indices are mutually exclusive, i.e. allogrooming was not counted as affiliative behavior, and passive close proximity and stationary vicinity were only recorded in the absence of other social interactions between two individuals.

^a^Follow is defined as following another individual by moving beside or behind with occasional physical contact.

The index values per individual are expressed as proportions for all four indices. With respect to affiliative behaviour and allogrooming, we considered the direction of the behaviour by calculating the percent of scans an individual spent with exhibiting affiliative behaviour towards an individual group member and grooming of a group mate, respectively. Since stationary vicinity and passive close proximity are symmetric, the index values are the same for the two interacting individuals in that case. Calculations are based on the number of scans the two interacting individuals had access to each other. Access to each other means that both individuals had access to the outdoor enclosure, which includes the scans where both individuals were in the outdoor enclosure, but also scans where one of the two individuals was indoors and thus not visible to the observer (access to indoor area depended on care decisions typically related to the weather conditions). We did consider total observation time per dyad because it could vary between the different dyads of a group as some individuals could have been separated for veterinary or care-management purposes or voluntarily stayed inside without access to the outdoor enclosure for periods of time.

For the multiplex analysis, in order to avoid an influence of layers on multiplex measures due to scaling effects, we normalized the index values of all four indices by dividing the individual values by the maximum value recorded for the respective layer (i.e. the highest value that occurred in one of the two groups). These values (= weighted index values) ranged from 0 (for a none existing edge) to 1 (representing the maximum index value layer) for all four indices then. In the multilayer analysis, within the MuxViz environment, the two social groups were analysed separately.

For statistical analysis in R (linear mixed models), we used the index values per individual for each social interaction type. The index definitions are the same as for the multilayer analysis. Here, all 14 individuals were analysed together when testing for effects of the biographic background on the indices. We considered the fact that the chimpanzees are living in two social groups by adding group as random effect and the ID of the sender as nested within group.

### Network construction

We used the MuxViz software^16^, in the R environment^57^, an open-source multilayer network visualisation and analysis software, for all network construction and exploration procedures presented in this study. We created a 4-layered multiplex network for each of the two groups of chimpanzees separately. All layers of the multiplex network were created as directed weighted networks, based on values ranging between 0 and 1, and all layers are interconnected by the nodes (representing the individuals) they have in common. Each layer contained only information of one of the four edge variables, as described in Table 2. As such, edges between two nodes reflect the existence and weight of a specific type of social interaction between them^58^.

### Social network analysis (SNA)

We applied several tools of network analysis offered by MuxViz:

#### Graphical visualisation

The MuxViz software offers a wide range of possibilities to graphically explore and represent social networks^16^. As visual representations help to detect trends or tendencies, we produced social networks for all layers and for the two social groups separately. We will also present annular visualisations of the node properties and layer rankings.

#### Interlayer correlation and reducibility

We examined the structural similarities between the four layers by inspecting the interlayer correlations in terms of edge-overlap. For testing structural similarities the sum of the weights of all edges connected to a node are considered by taking into account the fractions of edges shared between all four layers^16,59^.

In the next step, we applied the MuxViz reducibility analysis, based on the Von Neumann entropy, where the semi-aggregated states of multilayer networks are compared with the completely aggregated form. At each step of the algorithm, a multilayer network with one layer less is generated by aggregating the two most similar layers, i.e. the two layers with the smallest value of the quantum Jensen-Shannon divergence (Ward method for hierarchical clustering). Layers in which nodes are connected more similarly have a shorter Jensen-Shannon distance with a value closer to 0, whereas layers with very different connection patterns of nodes have values close to 1^20,21^. Since our multiplex networks are based on four layers, we reached the fully aggregated state of our networks after three steps of this merging procedure.

The fact that the reducibility analysis is based not only on the amount of connections but also the weights of each layer enabled us to apply this methodology even for small and densely connected networks where differences are more likely to occur due to the weights.

#### Node centrality/versatility

MuxViz offers a range of node measures for monolayer (centralities) as well as multilayer (versatilities) analysis^60–62^. We chose to calculate the eigenvector centrality and versatility to measure the importance of group members within a layer and between the layers within each of the two groups^63,64^. Eigenvector centrality is particularly suited for densely connected and small networks, as often found in primates^65^, as it accounts for edge weights, where more differences between individuals can be found compared to degree based centralities. This centrality measure considers the degree and strength of direct connections, but also takes indirect connections into account^62,66,67^. Each individual obtains a value between 0 (disconnected) to 1 (most densely connected) in each layer, the aggregate and multiplex state, which are than ranked accordingly. This allows us to compare the ranking position of the chimpanzees in a certain layer to their ranking positions in the other layers, the aggregate and multiplex states.

### Linear mixed models

For this part of the analysis, we included all 14 chimpanzees, but considered that they are living in two social groups. To investigate possible effects of the early life experience of our chimpanzees on the social interaction types (edges), we ran four linear mixed models (LMMs) with each of the edge variables (i.e. the index values) as dependent variable (Table S5). We ran a fifth linear mixed model with the aggregated values, calculated as the sum of the four edge variables, as dependent variable. All models were run by using the "lme4" package^68^ in R 3.5.0^57^.

In our recently published long-term study^30^, covering data from April 2006 to July 2018, we found allogrooming to be affected by predominant housing conditions during infancy (with or without conspecifics), origin (wild-caught vs. captive born) and sex (male vs. female), but not age (although wild-caught individuals were on average older than captive-born ones). We used the same fixed effects in this study to test for their effects on the four different social interaction types. Predominant housing condition during infancy (PHCinfant) considers if the chimpanzees were housed for more than 2.5 years of their first five years of life with or without conspecifics. With respect to predominant housing conditions during infancy and origin, we differentiated whether the individual directed the behaviour to a group member with the same experience or to a group member with a different experience. This resulted in four categories for predominant housing conditions during infancy (with- > with, without- > without, with- > without, without- > with) and origin (wild- > wild, captive- > captive, wild- > captive, captive- > wild). The same differentiation was done for sex (M- > M, F- > F, M- > F, F- > M). As the 14 chimpanzees live in two social groups, we included group as random factor and the ID of the sender of the behaviour as nested within group. We visually checked QQ plots for a normal distribution of the residuals (Figures S1 and S2). Fixed factors were the same in all LMMs, only the dependent variable differed for each model (i.e. the four edge variables and the aggregated value).

First, we tested whether full models (containing all three fixed factors) were significant improvements over the null models (without fixed factors). In case a full model differed significantly from the corresponding null model, we applied the ANOVA function (Type III Analysis of Variance with Satterthwaite´s method) and a post hoc test based on the p-value obtained with the “glht” function (multiple comparison of means with Tukey Contrast, p-values adjusted by the Holm-Bonferroni method). We tested for multicollinearity between all fixed factors by calculating the variance inflation factor (VIF) using the "car" package in R^69^. All VIFs (variance inflation factor), calculated for our three fixed factors were below 1.2, indicating that our fixed factors were not correlated.

## Results

### Graphical visualisation of the 4-layered multiplex networks

**Figure 1:**
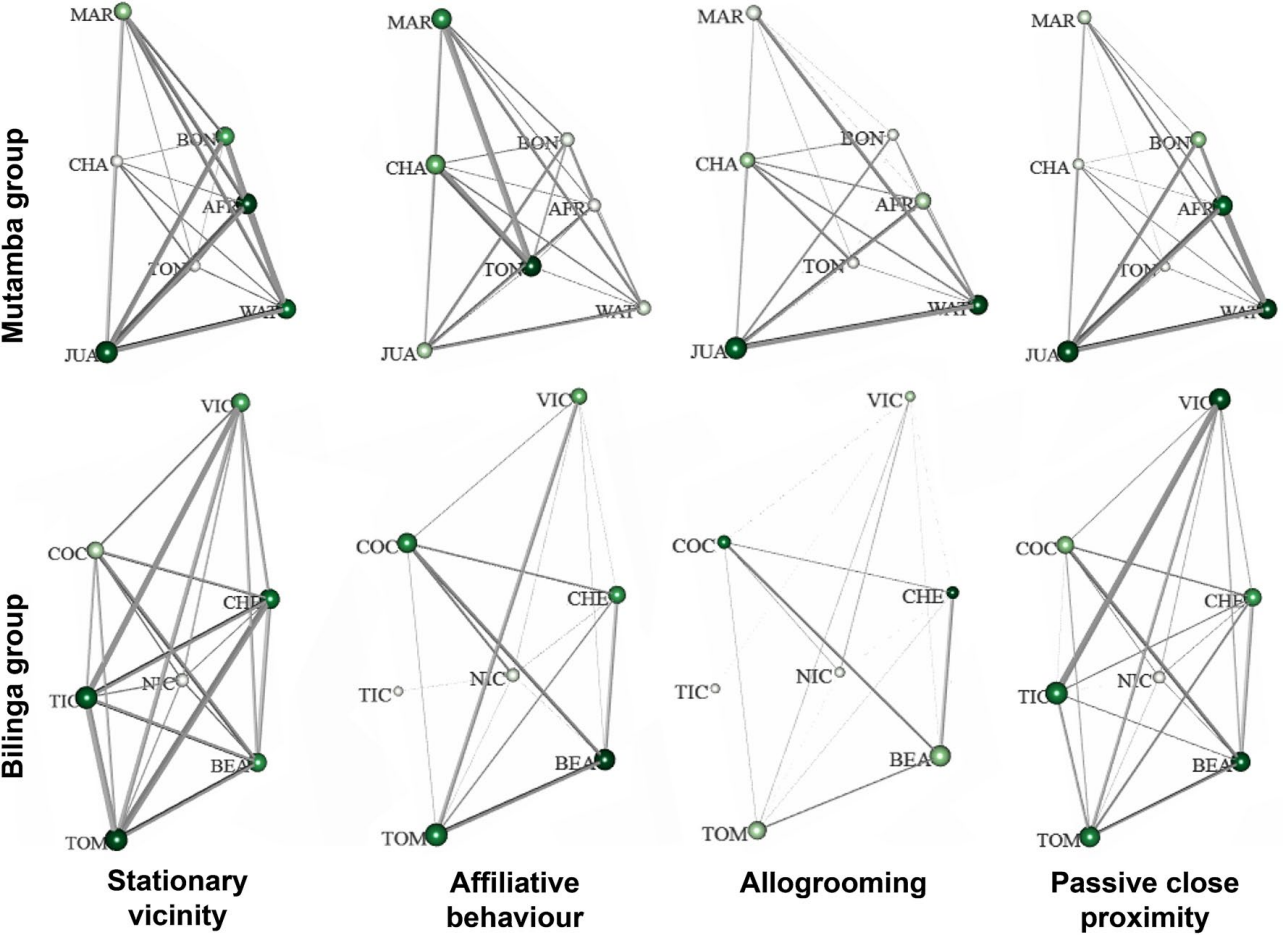
Multiplex networks of the two social groups (Mutamba and Bilinga). Each layer represents one of the four different social interaction types (stationary vicinity, affiliative behaviour, allogrooming and passive close proximity). Edge width is proportional to the directed weighted index value of node pairs. Node size is proportional to the eigenvector centrality. Node colours depend on the individual’s strength centrality. Node labels correspond to the individuals listed in Table 1. The node layout is based on the force-directed algorithm Kamada-Kawai to the aggregated network of all four layers, nodes have the same position on all layers.

The visual representation of each layer of the 4-layered multiplex networks for two groups of chimpanzees is shown in Fig. 1. The edges of the respective layers represent the particular social interaction types (stationary vicinity, affiliative behaviour, allogrooming, passive close proximity). The nodes represent the individuals of the respective group. The size of the nodes is based on the eigenvector centralities of the individual chimpanzees, i.e. the bigger a node the more *densely* is the respective individual connected to its group mates. The colour of the nodes refers to the strength centralities of the individual chimpanzees, i.e. the darker green a node the more *strongly* is the respective individual on average connected to its group members. The distribution of the nodes is based on the Kamada-Kawai algorithm. To give an example, female Cheeta (CHE) of the Bilinga group is strongly connected in all four interaction types as indicated by the darker green shaded node colour. However, she is not densely connected in the allogrooming layer, representing her eigenvector centrality, indicated by her small node size. For each of the two groups, each network of every single layer consists of seven nodes, representing the seven individuals (who are all in both groups present in all four layers). Thus, the 4-layered multiplex network has 168 possible edges for each of the two groups, of which 139 edges (i.e. 83%) are expressed in the Bilinga group and 166 edges (i.e. 99%) in the Mutamba group.

The present edges of the 4-layered multiplex network result in a network density of 0.83 for Bilinga and 0.99 for Mutamba, indicating densely connected networks for both groups. The network densities of the individual layers, however, are ranging from 0.57 to 1 (Table S1). The stationary vicinity layer had a network density of 1 in both social groups, i.e. all individuals spent some time out of an arm’s reach but within 5 m distance to all their group members. While affiliative behaviour was exchanged within all dyads of the Mutamba group, this was not the case in the Bilinga group where more than 25% of the possible edges were missing in this layer. The allogrooming layer had a network density of 0.57 in Bilinga group and 0.95 in Mutamba group, which means that allogrooming has been exchanged only in about half of the possible combinations in Bilinga group but in almost all combinations in Mutamba group. The passive close proximity layer had a network density of 1, again in both groups, i.e. all individuals spent some time within an arm’s reach to all their group members.

Network density (Table S1) also revealed that Mutamba group is more densely connected in three out of the four layers compared to Bilinga group. Stationary vicinity, representing a low-level social interaction type in terms of sociability, occurred much more often than the three other social interaction types, which are representing medium- to high-level social interaction types. Mean index values and mean weighted index values of the stationary vicinity layer were similar in both social groups, though the Mutamba group scored higher in all four interaction types. Affiliative behaviour, representing a medium-level social interaction type, occurred least frequently. Mean index values and mean weighted index values of the affiliative behaviour layer were again similar in both social groups. While the mean index values of the two social groups were similar for the close proximity layer, the two groups did differ in the allogrooming layer where we found a two times higher mean index value in the Mutamba group compared to the Bilinga group. Moreover, the comparison of the edges revealed that the individuals of the Bilinga group were much more selective with respect to allogrooming and affiliative behaviour than were the individuals of the Mutamba group.

### Interlayer correlation and layer reducibility

**Figure 2:**
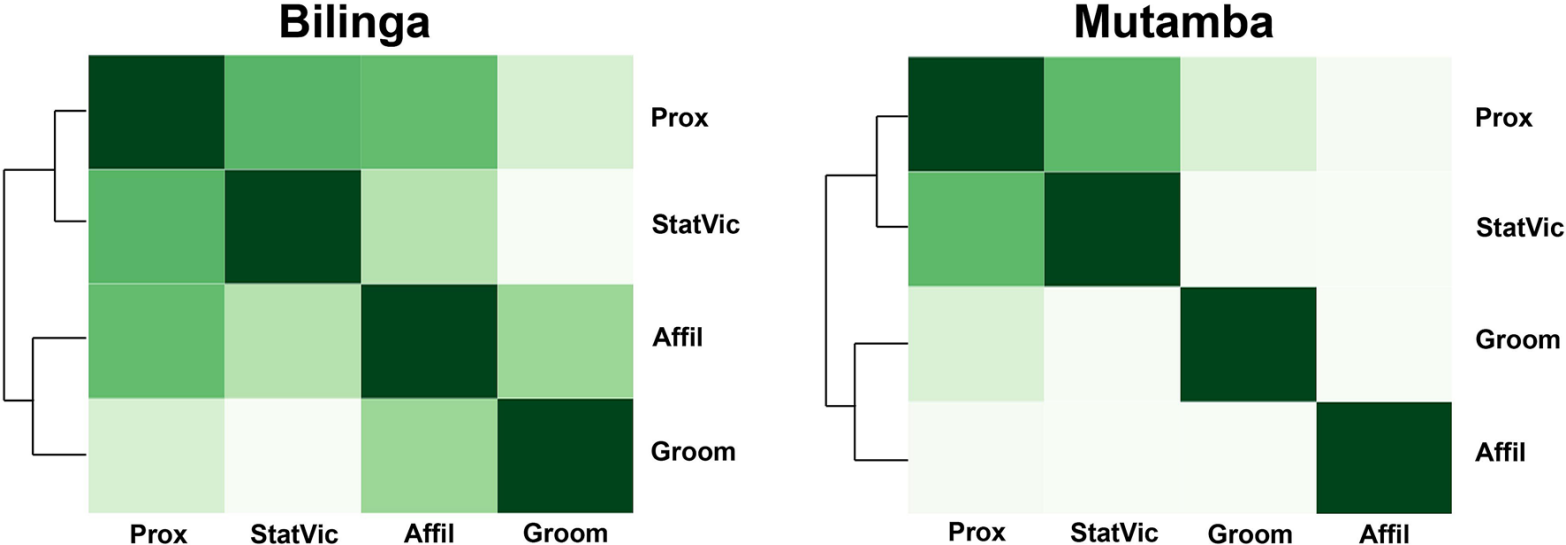
Interlayer differences evaluated via the edge-overlap between layers by detecting the fraction of edge values shared between all two-layer combinations, separately for the two social groups. Darker tones indicate a higher edge-overlap.

In order to evaluate differences and similarities between layers, we looked (1) into the overlapping of edges and (2) conducted a reducibility analysis. The overlapping of edges (reflecting the social interaction types) is presented in Fig. 2. The interlayer similarity is indicated by a dendrogram and the tone of the squares, i.e. the darker a tone the more similar are the respective layers.

The mean global edge-overlap (i.e. representing the fraction of edges, which are found in all four layers) is 10 percent for Bilinga group and 19 percent for Mutamba group (Table S2).

Figure 2 shows that the stationary vicinity layer and the close proximity layer are most similar to each other in both social groups (edge-overlap of 65% for Bilinga and 79% for Mutamba). In Bilinga group, the affiliative behaviour and the close proximity layer ranked second with an edge-overlap of 62%, followed by the layers allogrooming and affiliative behaviour with an overlap of 49%. The lowest edge-overlap was found between the layers allogrooming and stationary vicinity with 17%. In Mutamba group, the allogrooming and the close proximity layer ranked second with an edge-overlap of 61%, while all the other layer combinations had an overlap of 54%. In sum, it became apparent that individuals who were frequently in stationary vicinity to their group members were also often in close proximity to them in both social groups. There are, however, differences with respect to close proximity and allogrooming where we found a high edge-overlap in the Mutamba group but a low edge-overlap in Bilinga group indicating that individuals in Bilinga group who spent more time in close proximity to their group mates did not also spent more time grooming these group mates.

**Figure 3:**
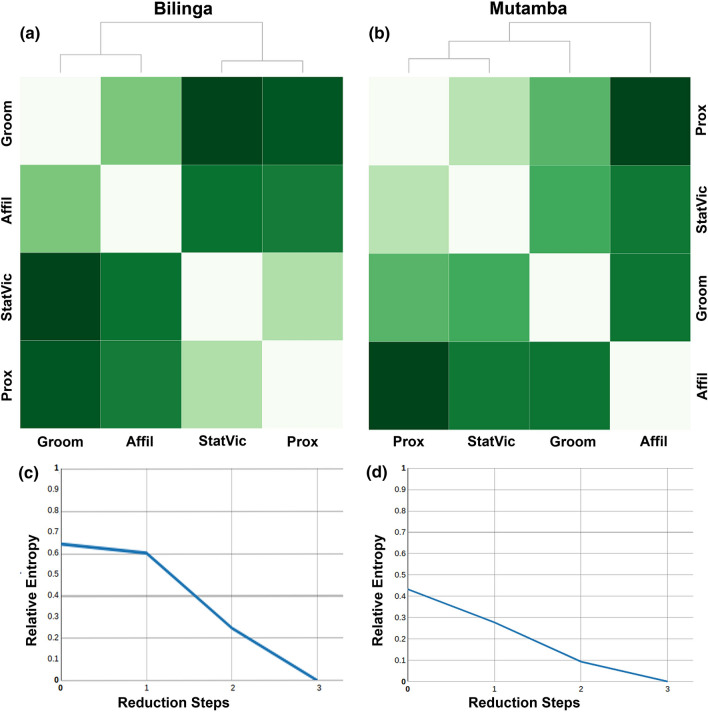
Visual representation of the reducibility analysis. Reducibility distance table for (**a**) Bilinga group and (**b**) Mutamba group. Layer-aggregation and network reducibility for (**c**) Bilinga group and (**d**) Mutamba group. In (**a**, **b**) layers have been sorted through a hierarchical clustering process using the Ward method with the dendrograms depicting the order of similarities. Darker tones indicate a greater distance (dissimilarity) between layers. (**c**,**d**) Present the relative entropy at each reduction step when comparing the 4-layered multiplex network with its respective semi- and fully aggregated network versions. At each step, the pair of layers with the shortest Jensen-Shannon distance (see **a**, **b**) is aggregated, reducing the number of layers by one. For both social groups the highest value of the relative entropy is reached in 4-layered multiplex network.

For the reducibility analysis, the interlayer similarity is calculated by the quantum Jensen-Shannon divergence, which estimates the similarity between two networks based on their Von Neumann entropy. Then a hierarchical clustering is performed by using the Ward method (Fig. 3a,b; Table S3). The dendrogram and the tone of the squares indicate the similarity of layers. Note that here a lighter tone indicates a higher similarity between layers. This is another measure to evaluate (dis-)similarities between layers where gradually the two layers with the shortest Jensen-Shannon distance (i.e. the most similar) are aggregated to one layer.

The reducibility analysis revealed—as seen before in overlapping of edges—that the stationary vicinity layer and the close proximity layer shared most similarities in both groups (Jensen-Shannon distances for Bilinga is 0.117 and for Mutamba is 0.102). In the Bilinga group, the allogrooming and the affiliative behaviour layer were next similar (0.172), followed by the affiliative behaviour and the close proximity layer (0.291). The highest dissimilarity was found for the allogrooming and the stationary vicinity layer (0.358). This ranking (Table S3) is almost the same as the one found when comparing the overlapping of edges.

In the Mutamba group the allogrooming layer and the close proximity layer ranked second with respect to their similarity (0.197) consistent to the finding in the overlapping of edges. Compared to the edge-overlap where all the other layer combinations ranked the same, these layer combinations ranked different here. The greatest dissimilarity was found between the affiliative behaviour and the close proximity layer (0.344; Table S3).

Additionally to the visual presentation, the relative entropy is calculated for every reduction step (Fig. 3c,d). For both social groups the reducibility analysis clearly revealed that each layer aggregation step leads to a loss of information indicated by a decreasing relative entropy. This shows that the 4-layered multiplex networks are the most optimal representation in both social groups.

### Node centralities and versatilities

**Figure 4:**
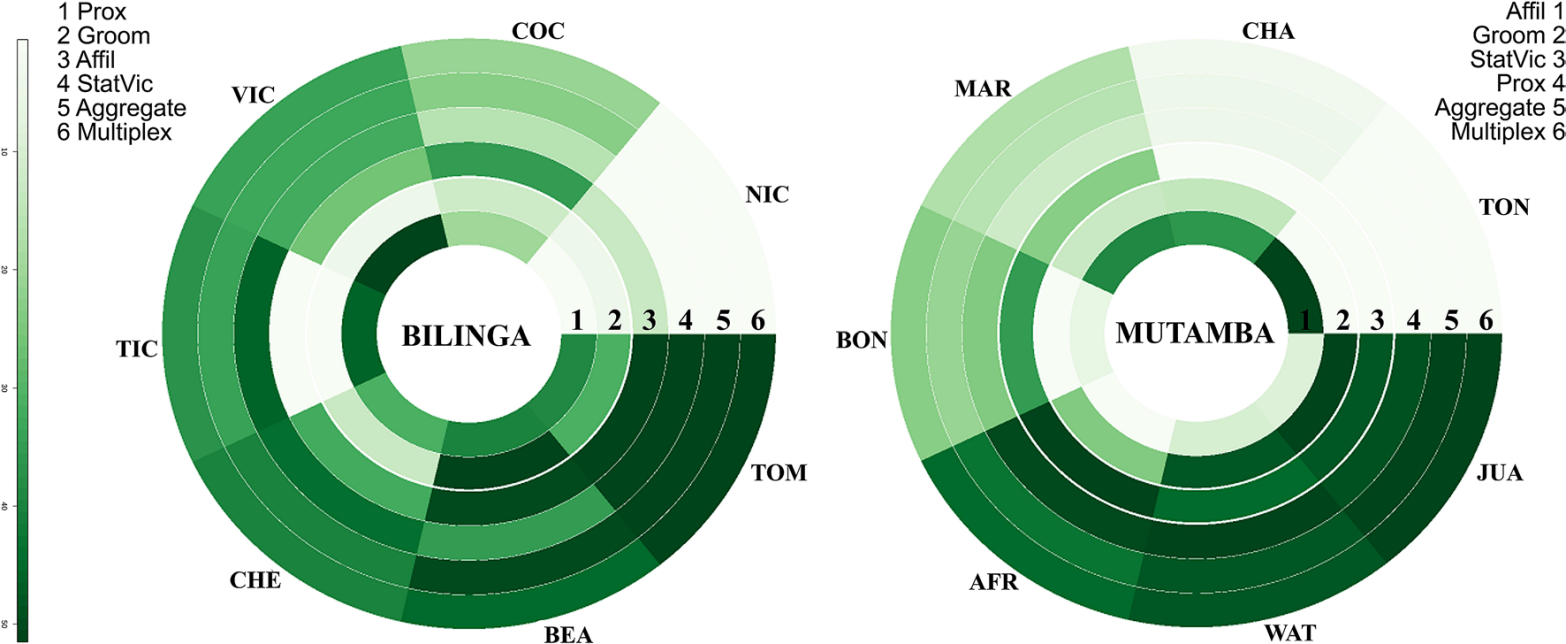
Annular visualisation of the eigenvector centralities and versatility for both chimpanzee groups (Bilinga, Mutamba). Eigenvector values are colour-scaled with darker tones representing higher values according to the scale on the left side. Each ring represents either a single layer, an aggregated layer or the multiplex network. Note that the ring order is different for the two groups (numbering of the rings refers to the respective legend). Each triangle shaped segment, cutting across all six rings, represents the eigenvector values of one particular chimpanzee. The order of the segments is based on the versatility rank of the individuals (clockwise order of the eigenvector versatility from highest to lowest), i.e. ordering of Bilinga group is based on ring 1 (inner ring), ordering of Mutamba group is based on ring 6 (outer ring).

For the annular visualisation we calculated the eigenvector centrality (for each layer and for the fully aggregated network where all four layers are aggregated to a single layer) and the eigenvector versatility for each node (individual). In this visualisation (Fig. 4), each ring represents the eigenvector centralities of a single layer and the aggregated layer as well the eigenvector versatility of the multiplex network. For differences between the aggregated layer and the multiplex network, see Solé-Ribalta et al.^70^. The order of the rings is based on the similarity ascertained by a Spearman correlation. Each triangle shaped segment represents the eigenvector values of one individual where darker tones indicate higher values. The order of the individuals is based on the individuals’ versatility rank (see Table S4).

The annular visualisations of the eigenvector centralities and versatilities of the two social groups provide an insight not only on the layer level but also on the individual level. Higher eigenvector centralities, indicated by a darker tone, refer to the relative importance of the respective individual. It becomes apparent that some individuals such as Juanito, Waty and Africa in the Mutamba group, and Tom and Bea in the Bilinga group are more sociable than other ones as they scored high in almost all layers. Whereas individuals such as Nico in Bilinga group, and Charly and Toni in Mutamba group seem less sociable than their group members as they scored low in almost all layers. On closer inspection, however, it becomes obvious, that the supposedly less sociable individuals Charly and Toni scored very high in the affiliative behaviour layer. This explains also our findings from edge overlapping and the reducibility analysis. There we found in the Mutamba group the affiliative behaviour layer to be most dissimilar from the stationary vicinity and the close proximity layer. Similarly, the finding of the greatest dissimilarity between the allogrooming layer and the close proximity layer can be explained by Victor and Tico who scored very low in the allogrooming layer but very high in the close proximity layer. This shows that sociability can only be reliably estimated by taking several different interaction types into account.

### Linear mixed models

Since the annular visualisation provided an indication of individual differences, we ran linear mixed models where we considered the biographical background of our individuals. We found three out of the five full models to show significant improvements compared to their respective null models. The full models with stationary vicinity and the aggregated variable as dependent variables showed no improvement compared to the null model. All outcomes of the three LMMs and the respective post hoc analyses are presented in the supplementary Tables S5–S6.

**Figure 5:**
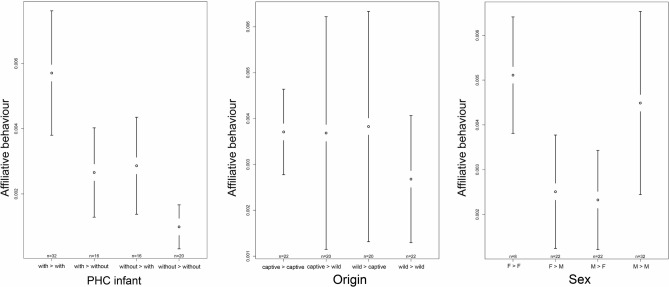
Confidence interval plots of affiliative behaviour and the three fixed effects predominant housing condition during infancy (PHC infant), origin and sex. Mean index value over all directed dyads (± 95% CI).

The full model with affiliative behaviour as dependent variable revealed a significant effect of origin (F = 4.272, p = 0.007), predominant housing condition during infancy (PHCinfant; F = 12.447, p < 0.001) and sex (F = 2.892, p = 0.040) on the occurrence of affiliative behaviour (see Table S5). With respect to origin, we found wild-caught individuals to exhibit significantly more affiliative behaviour toward captive born individuals and captive born individuals to exhibit significantly more affiliative behaviour toward wild-caught individuals compared to the affiliative behaviour exhibited between two captive born individuals (captive- > wild vs. captive- > captive: z = 3.120, p = 0.009; wild- > captive vs. captive- > captive: z = 3.184, p = 0.009; Fig. 5 and Table S6). With respect to predominant housing condition during infancy, affiliative behaviour was shown significantly more often between individuals who were both predominantly housed with conspecifics compared to the other combinations (with- > without vs. with- > with: z = −4.328, p < 0.001; without- > with vs. with- > with: z = − 4.193, p < 0.001; without- > without vs. with- > with: z = − 5.967, p < 0.001; Fig. 5 and Table S6). Regarding sex, we found females to direct significantly more affiliative behaviour toward females than toward males (F- > M vs. F- > F: z = − 2.590, p = 0.048), and males to direct significantly less affiliative behaviour toward females than females toward females (M- > F vs. F- > F: z = − 2.673, p = 0.045; Fig. 5 and Table S6).

**Figure 6:**
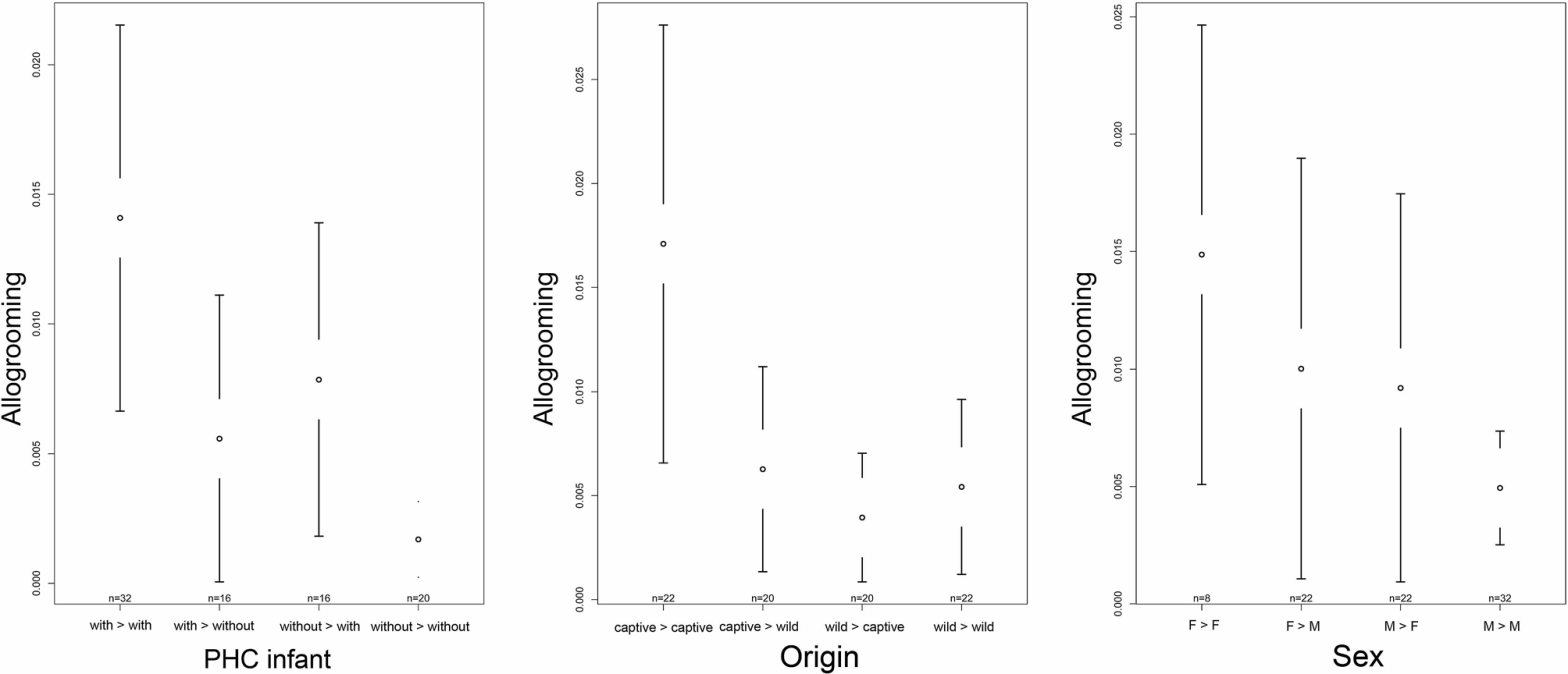
Confidence interval plots of allogrooming and the three fixed effects predominant housing condition during infancy (PHC infant), origin and sex. Mean index value over all directed dyads (±95% CI).

In our full model with allogrooming as dependent variable we found a significant effect of origin (F = 3.085, p = 0.032) and sex (F = 6.032, p < 0.001) on the time spent allogrooming (see Table S5). Predominant housing condition during infancy did not significantly affect time spent on allogrooming. However, there was a trend showing that grooming was exchanged less frequently among individuals who were housed predominantly without conspecifics compared to the exchange of grooming among individuals who were housed predominantly with conspecifics during infancy (without- > without vs. with- > with: z = − 2.485, p = 0.078, Fig. 6 and Table S6). With respect to origin, we found wild-caught individuals to spent significantly less time grooming their wild-caught group mates compared to the time captive born individuals spent grooming their captive born group members (wild- > wild vs. captive- < captive: z = − 2.904, p = 0.022; Fig. 6 and Table S6). A trend became apparent when comparing the time wild-caught individuals spent grooming their captive born group members and the time captive born individuals spent grooming their captive born group mates (wild- > captive vs. captive- > captive: z = − 2.501, p = 0.062). Regarding sex, we found males to spend significantly less time grooming other males than females (M- > M vs. M- > F: z = − 2.630, p = 0.034). Males groomed each other also significantly less often than females groomed each other but also males (M- > M vs. F- > F: z = − 3.783, p < 0.001; M- > M vs. F- > M: z:− 3.092, p = 0.010; Fig. 6 and Table S6).

**Figure 7:**
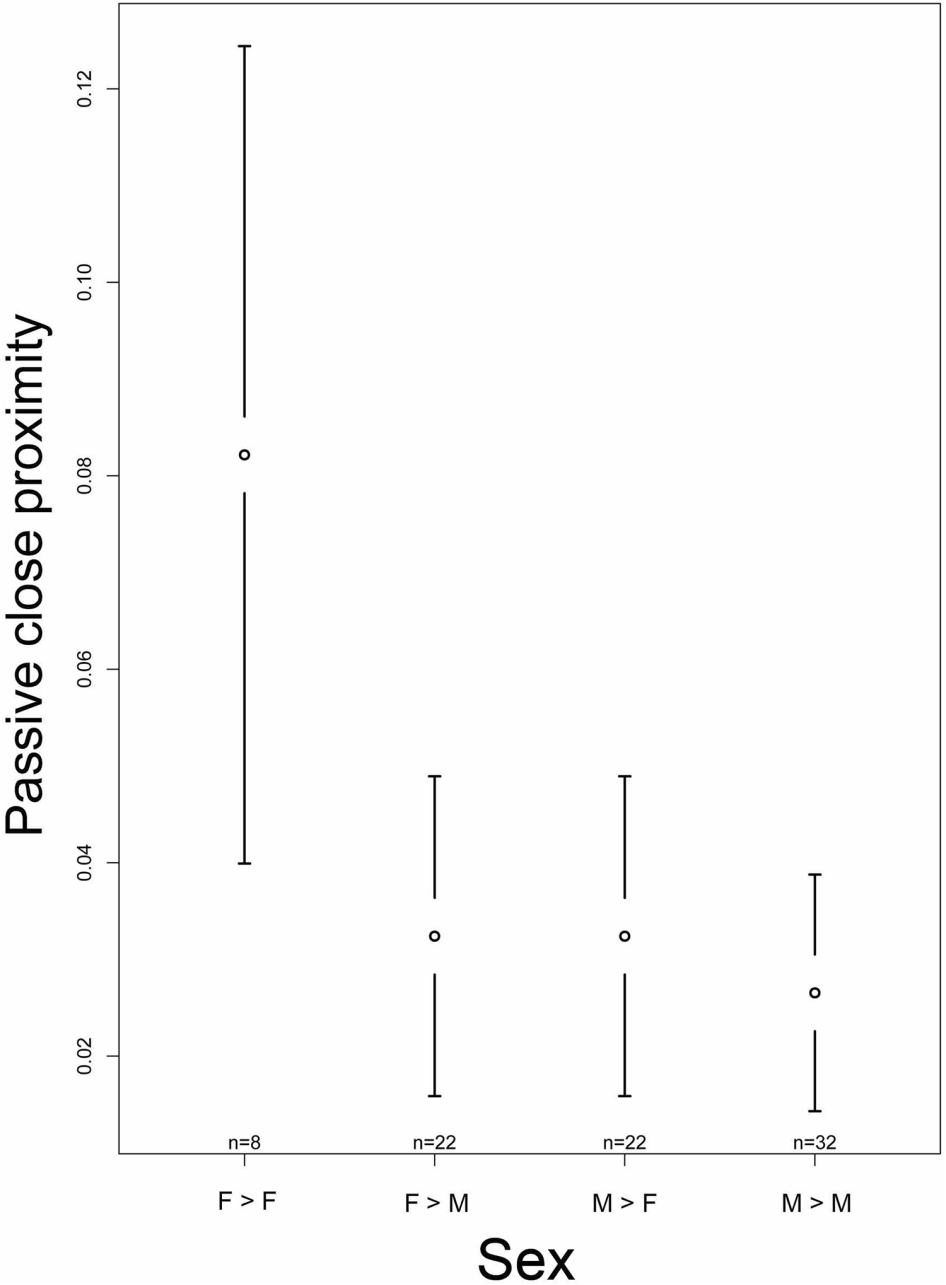
Confidence interval plots of passive close proximity and the two fixed effects origin and sex. Mean index value over all dyads (±95% CI).

The full model with passive close proximity as dependent variable revealed a significant effect of sex (F = 6.527, p < 0.001) on the time spent in passive close proximity (see Table S5). Predominant housing condition during infancy and origin did not significantly affect passive close proximity. With respect to sex, we found females to spend significantly more time in close proximity to females than to males (F- > M vs. F- > F: z = − 4.003, p < 0.001). Males spent significantly less time in close proximity to males but also to females than females spent in close proximity to females (M- > M vs. F- > F: z = − 4.142, p < 0.001; M- > F vs. F- > F: z = − 3.656, p = 0.001; Fig. 7 and Table S6).

In sum, we found sociability, reflected in affiliative behaviour and allogrooming, to be affected by predominant housing conditions during infancy and/or origin as well as sex. Affiliative behaviour as well as allogrooming were most frequently exchanged among individuals who were both predominantly housed with conspecifics during infancy. In addition, allogrooming was exchanged most frequently among captive born individuals and least frequently among wild-caught individuals. Sex was the only fixed factor that was consistently affecting the depended variables in all three models, where females had the highest values in their exchange of affiliative behaviour as well as allogrooming among each other and spent most time in close proximity to each other compared to the other combinations.

## Discussion

The multiplex approach revealed that it is possible to consider different social interaction types simultaneously even in small social groups of chimpanzees, i.e. with a group size of seven individuals. We did find similarities but also differences between the four social interaction types stationary vicinity, affiliative behaviour, allogrooming and passive close proximity, which in turn increased the information gain by giving insights into the sociability of these two groups of former pet and entertainment chimpanzees. By taking into account the early life history of interaction partners and doing so in two social groups with different group composition regarding said early life experiences, we could detect certain differences in the occurrence of social interaction types.

Although admittedly the data collection and preparation has been more complex and extensive, by applying the multiplex approach, we were able to conduct a far more realistic social network representation and analysis. Looking at the (dis-)similarities between layers and eigenvector rankings across the interaction types, none of the four individual layers on their own provides insights representing the information from the remaining layers. While the aggregate layer would have at least assured that chimpanzees scoring low in one particular layer would not have been marked automatically as an outsider or individual with a low sociability, it still failed in our secondary objective related to the early life experiences of the interaction partners. According to our results, at least for small networks such as ours, the aggregate layer might be a better option than a single layer network, but is prone to lose much information content as demonstrated in the reducibility analysis and LMM analysis regarding the atypical life history.

A general overview on the (dis-)similarities of the four social interaction types was provided by investigating the overlapping of edges. There it became apparent that individuals who spent more time in stationary vicinity, i.e. out of an arm’s reach but within 5 m of their group mates, also spent more time in passive close proximity, i.e. within an arm’s reach of their group members. This pattern was found in both social groups. A more detailed view on the similarity of the layers, i.e. the four social interaction types, and the information value of each layer is given by the reducibility analysis. The outcome of this analysis confirmed the edge-overlap finding on the similarity of the stationary vicinity and the passive close proximity layer. However, in addition, it also showed that in the Bilinga group there is high dissimilarity between stationary vicinity and allogrooming. Generally speaking, this means that individuals who spent more time in stationary vicinity to their group mates spent less time grooming their group mates and vice versa. In the Mutamba group, the highest dissimilarity was found between affiliative behaviour and passive close proximity, which means that there was a tendency that individuals who performed more affiliative behaviour toward their conspecifics spent less time in close proximity to them and vice versa. Since Bilinga group consists of a majority of wild-caught individual and Mutamba group of a majority of captive born individuals this already provides a first indication that the biographical background of the individuals may be important for these differences found in the two social groups. We will come back to this when discussing the outcome of the linear mixed models where we considered the individuals’ early life experience.

The reducibility analysis is, furthermore, beneficial in that it shows whether layers can be reduced without losing information. In our case, the analysis revealed that each layer provides information that would be lost by a reduction of layers. The most detailed view is given by the annular visualisations of the eigenvector centralities and versatilities, which measure the importance of group members within a layer and between the layers, and the graphical visualisation of the multiplex networks. The annular visualisation allows a direct comparison of the eigenvector values of the social interaction types per individual as these values are ordered in rings. In our case, this visualisation revealed that some individuals scored high in almost all social interaction types and some individuals scored low in most of the social interaction types. That means that some individuals are much more sociable than other ones. However, this annular visualisation also indicates that sociability can only be reliably accessed by looking at different social interaction types simultaneously as some individuals scored low in some social interaction types but high in other ones. A different way of representation is the visualisation of the multiplex network where information on not only the strength, but also the density (eigenvector centrality) of the connection of every individual of a group is shown. Here it became obvious as well, that some individuals are more strongly and densely connected to their group mates in most of the social interaction types than are other ones.

This is why we conducted linear mixed models in addition to find out whether the biographic background of our chimpanzees might at least partly explain these differences found and thus emphasise the benefit of taking several interaction types into account. We considered the origin of the interaction partners, i.e. whether they were caught from the wild or born in captivity, the predominant housing condition during their infancy, i.e. whether they were housed more than 2.5 of their first five years of life with or without conspecifics, and the sex. Thus, we differentiated if the sender directed the behaviour to a conspecific with the same experience or to a group mate with a different experience as we expected some flexibility in the behaviour of captive born and/or predominantly socially housed individuals. Whereas we expected wild-caught and predominantly singly housed individuals to be more impaired by their adverse early life experience and accordingly to be more rigid in their behaviour, especially with respect to medium- to high-level social interactions types such as affiliative behaviour, allogrooming and passive close proximity.

Indeed, we found the effects of early life experience to be detectable in certain social interaction types. Affiliative behaviour was significantly more often exchanged within dyads where both individuals were predominantly housed with conspecifics during their infancy compared to the other dyadic combinations. It occurred least often within dyads consisting of two individuals who were both predominantly housed without conspecifics during infancy. The same trend was found for allogrooming, though it did not reach significance. This findings stress the importance of social learning, especially during infancy, which requires an appropriate social environment including the mother and peers among other group members^23,71–73^. Atypically reared chimpanzees lacking tactile stimulation during infancy may not experience the tension-reducing and relaxing effects of allogrooming^74,75^ but may find physical contact rather stressful, which would be reflected in an avoidance of grooming activities.

Affiliative behaviour and allogrooming were both significantly affected by origin as well. Interestingly, affiliative behaviour occurred most frequently in captive born individuals towards wild-caught group mates and vice versa, whereas allogrooming was exchanged most often within dyads consisting of two captive born subjects and least often within dyads composed of two wild-caught individuals. This pattern implies that captive born individuals adjust their behaviour to their vis-á-vis. While they exchange allogrooming, a high-level social interaction type with each other, they switch to the exchange of a medium-level social interaction type – affiliative behaviour including social play and follow – when interacting with wild-caught group mates. This is in line with findings in ex-laboratory chimpanzees where later deprived individuals compensated the lack of social grooming of early deprived conspecifics by time spent on gentle social play with them^50^. We believe that these results provide an indication of the significance of considering the sociability of the individuals when composing groups, which among other factors is influenced by the individuals’ early life history. Socially functioning groups are one key factor to ensure the wellbeing of individuals who are cared for in captivity.

Unexpectedly, we did not find any effect of origin and/or predominant housing conditions during infancy on the toleration of passive close proximity, which we rated as high-level social interaction type. This outcome is in contrast to the results found in early deprived ex-laboratory chimpanzees who were living in solitary confinements for decades before being re-socialised compared to their later deprived conspecifics^50^ but in line with findings on wild-caught zoo chimpanzees who had been socially reared in comparison to maternally and socially reared captive born individuals^26^.

With respect to our wild-caught individuals, it is known that there are long-lasting outcomes of childhood trauma reflected in an impaired social adjustment not only in chimpanzees^46^ but also in humans^76^. However, we did not find our wild-caught chimpanzees to be unable to perceive their social environment as safe and their conspecifics close by as not thrust-worthy^77^ as this would have been reflected in an avoidance of close proximity. The impairment of our adult wild-caught chimpanzees and those who grew up without conspecifics with respect to allogrooming may also be based on the lacking stimulation and arousal modulation experienced during early infancy^78^. Bründl et al.^79^ mapped the development of social interaction and communication traits in a longitudinal sample of wild chimpanzees and found the emergence of social interactions at a mean age of 14 months, with mutual grooming not occurring before around 38 months of age. These findings reaffirm that the first years of life are a crucial period in a chimpanzee’s development.

Returning to our hypotheses concerning the multiplex network analysis, where we expected to find correlations between 1) medium- to high-level social interactions types that require the toleration of body contact, i.e. between affiliative behaviour and allogrooming, and 2) high-level social interaction types in terms of sociability, i.e. allogrooming and passive close proximity, the following can be said. We could confirm our first hypothesis as we found a high similarity between affiliative behaviour and allogrooming, social interaction types that both require at least some physical contact, but not our second hypothesis as we found a high dissimilarity between allogrooming and passive close proximity. Contrary to our expectation, we found the highest similarity between stationary vicinity and passive close proximity. With respect to the effects of the biographical background of our chimpanzees on the different interaction types, we found origin and/or predominant housing conditions during infancy to affect affiliative behaviour and allogrooming but not passive close proximity. Beyond that, the sex of the chimpanzees had an effect on affiliative behaviour, allogrooming and passive close proximity. Although studies on wild-living chimpanzees found social interactions to be more frequent among males than females, Lehman and Boesch^80^ suggested that this is mainly caused by the habitat conditions including food availability and dispersal patterns. With these factors controlled for by captive management decisions and competition for resources being less of an issue in captivity, the social potential of female chimpanzees becomes apparent^81^. Thus, we were not surprised that our results indicated that social interactions were most frequent within female-female dyads and occurred less frequently within male-male as well as within mixed-sex dyads.

The fact that we found early life experience to have an effect on medium- to high-level social interaction types in term of sociability does not rule out that other factors such as personality^82,83^ may play a role as well. Moreover, we do expect an at least partial recovery in a nurturing environment as provided by a rescue centre^84^.

We think that the full potential of the multiplex analyses can be utilised when used for the investigation of larger social groups of primates as has been done with a group of free-living Geoffroy’s spider monkeys^18,19^. In case of captive managed groups of primates, the multiplex analyses would for example allow to investigate the exchange of affiliative and agonistic behaviour simultaneously and, thus, to detect problematic relationships among certain group. It could also be used to realistically identify outsiders, where the assessment is based on more than one specific social interaction type, which as demonstrated might be an imperfect indicator for sociability on its own. In such a manner, the multiplex approach could be used as a kind of diagnostic tool, supporting care management decisions including decisions on alterations of groups. Furthermore, these multilayer network analyses would also allow to visualize and test complex exchanges of interactions such as whom helps social grooming to gain agonistic support^85,86^ and in whom is allogrooming reducing tension^75,87^.

In larger social groups, it would also be possible to take the biography of the individuals into account, e.g. whether the sender was maternally or hand-reared, and by creating separate layers for maternally reared senders and hand-reared senders the distribution of a certain behaviour could be directly compared. We therefore believe that the multiplex approach may be a helpful tool in the management of larger groups of primates in captivity.

In conclusion, it can be said, that the multiplex analyses are a useful tool for investigating the sociability of, as in our case, former pet and entertainment chimpanzees because different social interaction types can be considered simultaneously. Furthermore, the reducibility analysis allows testing for redundancy, i.e. whether different social interaction types provide an information gain or not. We believe that it is worthwhile to apply this multiplex approach even to small groups of primates, although small sized populations produce certain limitations as, for example, several tools, such as community structure analysis or triadic relationships, provided by the MuxViz software cannot be used when investigating small social groups. Furthermore, the algorithms that differentiate the simple aggregation layer from the multiplex state become relevant and useful in large scale networks with more scattered connections between nodes and layers (see Solé-Ribalta et al.^70^ for differences between the aggregated and multiplex state). The full potential of this multiplex approach could be utilized by applying it to large groups of primates where, e.g. individual characteristics such as the biographic background could be considered when comparing the different social interaction types. Unfortunately, this was not possible in our small groups consisting of seven chimpanzees. With long-term data collected over several years, it would also be possible to expand the number of layers by adding behaviours that occur less often as, for example, agonistic behaviour. By considering the direction of these behaviours, the exchange of affiliative and agonistic behaviour could be investigated simultaneously. Hence, the multiplex approach can be seen as a promising tool for the management of (larger) groups of primates housed in captivity as it allows to detect problematic relationships among certain group members and individuals who are not involved in any social interactions as all.

## Supplementary information

Supplementary Information 1.

Supplementary Information 2.
