# Supplementary material for: Assessing the sociability of former pet and entertainment chimpanzees by using multiplex networks
Source: Sci Rep. 2020 Dec 1;10:20969. doi: 10.1038/s41598-020-77950-x (PMC7708499; doi:10.1038/s41598-020-77950-x)
Supplement: Supplementary file 1 — Supplementary Information 1. [file 41598_2020_77950_MOESM1_ESM.docx]

**Assessing the sociability**

**of former pet and entertainment chimpanzees**

**by using multiplex networks**

Dietmar Crailsheim^1,2*^, Toni Romani^3^, Miquel Llorente^1,2,4^, Elfriede Kalcher-Sommersguter^5^

**1** Unitat de Recerca i Etologia, Fundació MONA, Riudellots de la Selva, Spain

**2** Facultat d’Educació i Psicologia, Universitat de Girona, Girona, Spain

**3** Faculty of Artes Liberales, University of Warsaw, Warsaw, Poland

**4** Institut de Recerca i Estudis en Primatologia - IPRIM, Girona, Spain

**5** Institute of Biology, University of Graz, Graz, Austria

**Supporting information**

**Supplementary Table S1.** Descriptive information on the 4-layered multiplex networks for the two social groups (Bilinga, Mutamba).

|  | **Multiplex layer** | **Mean index value** | **Min-max index value** | **Mean weighted index value** | **Min-max weighted index value** | **Nodes** | **Edges** | **Network density** |
| --- | --- | --- | --- | --- | --- | --- | --- | --- |
|  |  |  |  |  |  |  |  |  |
|  |  |  |  |  |  |  |  |  |
| **Bilinga group** | Stationary vicinity | 0.190 ± 0.120 | 0.047 - 0.465 | 0.399 ± 0.252 | 0.099 - 0.980 | 7 | 42 | 1 |
|  | Affiliative behaviour | 0.003 ± 0.003 | 0.000 - 0.010 | 0.150 ± 0.160 | 0.014 - 0.545 | 7 | 31 | 0.74 |
|  | Allogrooming | 0.006 ± 0.009 | 0.000 - 0.038 | 0.066 ±0.098 | 0.003 - 0.418 | 7 | 24 | 0.57 |
|  | Passive close proximity | 0.031 ± 0.031 | 0.001 - 0.133 | 0.196 ± 0.194 | 0.003 - 0.837 | 7 | 42 | 1 |
| **Mutamba group** | Stationary vicinity | 0.169 ± 0.143 | 0.031 - 0.475 | 0.357 ± 0.302 | 0.065 - 1.000 | 7 | 42 | 1 |
|  | Affiliative behaviour | 0.005 ± 0.005 | 0.001 - 0.018 | 0.264 ± 0.257 | 0.055 - 1.000 | 7 | 42 | 1 |
|  | Allogrooming | 0.014 ± 0.019 | 0.000 - 0.090 | 0.154 ± 0.213 | 0.002 - 1.000 | 7 | 40 | 0.95 |
|  | Passive close proximity | 0.039 ± 0.047 | 0.001 -0.159 | 0.243 ± 0.298 | 0.007 - 1.000 | 7 | 42 | 1 |

**Supplementary Table S2.** Interlayer edge-overlap. Fraction of shared edges between layers and mean global edge-overlap for both chimpanzee groups. Last column shows the average values over both social groups.

|  | **Mutamba** | **Bilinga** | **Group Average** |
| --- | --- | --- | --- |
| **Mean global edge-overlap** | **0.19** | **0.10** | **0.14** |
| **Groom - StatVic** | 0.54 | 0.17 | 0.36 |
| **Groom - Affil** | 0.54 | 0.49 | 0.51 |
| **Groom - Prox** | 0.61 | 0.32 | 0.47 |
| **Affil - Prox** | 0.54 | 0.62 | 0.58 |
| **Affil - StatVic** | 0.54 | 0.43 | 0.48 |
| **Prox - StatVic** | 0.79 | 0.65 | 0.72 |
| **STDEV** | **0.10** | **0.18** | **0.12** |
| **Average** | **0.59** | **0.45** | **0.52** |

**Supplementary Table S3.** Reducibility Analysis. The Jensen-Shannon distances are used for the hierarchical clustering of the multiplex layers. These values defined the order of the layer reduction process (for each social group). Values range from 0 to 1 with larger values representing a greater distance (difference) between layers.

|  | **Mutamba** | **Bilinga** | **Group Average** |
| --- | --- | --- | --- |
| **Prox - StatVic** | 0.102 | 0.117 | 0.110 |
| **Groom - Affil** | 0.289 | 0.173 | 0.231 |
| **Groom - Prox** | 0.197 | 0.338 | 0.268 |
| **Affil - Prox** | 0.344 | 0.291 | 0.318 |
| **Groom - StatVic** | 0.217 | 0.358 | 0.288 |
| **Affil - StatVic** | 0.285 | 0.304 | 0.295 |
| **Average** | **0.239** | **0.264** | **0.251** |
| **SD** | **0.086** | **0.096** | **0.091** |

**Supplementary Table S4.** Eigenvector centrality and versatility values of the social groups. First column refers to the individual. Columns 2-5 represent the Eigenvector centralities for each layer of the multiplex network separately. Column 5 is the calculated Eigenvector of a fully aggregated network version (where all 4 layers have been aggregated to one single layer). Column 7 represents the Eigenvector versatility (multiplex equivalent to the Eigenvector centrality) as described by De Domenico et al. ([2015](#_ENREF_1)). Column 8 represents the Versatility rank based on the eigenvector versatility (column 7). *Rows are ranked according to the versatility rank.* *Within the columns, values higher than the average are highlighted in green, values lower than the average are highlighted in in purple, with the highest value written bold.*

| **Bilinga** |  |  |  |  |  |  |  |
| --- | --- | --- | --- | --- | --- | --- | --- |
| **ID** | **Groom** | **Affil** | **Prox** | **StatVic** | **Aggregate** | **Multiplex** | **Versatility Rank** |
| TOM | 0.6106 | **1.000** | 0.8180 | **1.000** | **1.000** | **1.000** | 1 |
| BEA | **1.000** | 0.9924 | 0.8335 | 0.7753 | 0.9899 | 0.9263 | 2 |
| CHE | 0.2489 | 0.6364 | 0.6798 | 0.9013 | 0.8414 | 0.8470 | 3 |
| TIC | 0.0008 | 0.0042 | 0.9351 | 0.9286 | 0.7562 | 0.8068 | 4 |
| VIC | 0.0777 | 0.5079 | **1.000** | 0.7325 | 0.7488 | 0.7628 | 5 |
| COC | 0.2354 | 0.6991 | 0.5127 | 0.4932 | 0.6010 | 0.5639 | 6 |
| NIC | 0.0745 | 0.2624 | 0.1914 | 0.2664 | 0.2706 | 0.2657 | 7 |
| **Mutamba** | | | | | | | |
| **ID** | **Groom** | **Affil** | **Prox** | **StatVic** | **Aggregate** | **Multiplex** | **Versatility Rank** |
| JUA | **1.000** | 0.4410 | 0.9662 | 0.9669 | **1.000** | **1.000** | 1 |
| WAT | 0.9572 | 0.4561 | **1.000** | 0.9189 | 0.9599 | 0.9648 | 2 |
| AFR | 0.5668 | 0.3205 | 0.9821 | **1.000** | 0.8977 | 0.9163 | 3 |
| BON | 0.1735 | 0.3988 | 0.5211 | 0.7550 | 0.5866 | 0.6091 | 4 |
| MAR | 0.3766 | 0.8484 | 0.3020 | 0.5578 | 0.5172 | 0.5297 | 5 |
| CHA | 0.4011 | 0.8027 | 0.1461 | 0.2005 | 0.3387 | 0.3264 | 6 |
| TON | 0.1786 | **1.000** | 0.0835 | 0.1914 | 0.2936 | 0.2899 | 7 |

**Supplementary *Table S5.*** *Linear mixed models based on each interaction type and its fully aggregated state.*

|  |  |  | Anova Type III Analysis of Variance Table with Satterthwaite's method | | | | | | | | | | | |
| --- | --- | --- | --- | --- | --- | --- | --- | --- | --- | --- | --- | --- | --- | --- |
|  | | | **Origin** | | | | **PHCinfant** | | | | **Sex** | | | |
| **Model** | **∆ AIC** | **p** | **SumSq** | **Mean Sq** | **Fvalue** | **p** | **SumSq** | **Mean Sq** | **Fvalue** | **p** | **SumSq** | **Mean Sq** | **Fvalue** | **p** |
| **Groom** | 6.9 | 0.00309** | 0.0014498 | 0.00048325 | 3.0853 | 0.0316077 * | 0.0010059 | 0.00033531 | 2.1408 | 0.1011569 | 0.0028343 | 0.00094477 | 6.0319 | 0.0009025 *** |
| **Affil** | 15.3 | 0.0001209 *** | 0.00013734 | 4.5778e-05 | 4.2721 | 0.007391 ** | 0.00040013 | 1.3338e-04 | 12.4470 | 8.211e-07 *** | 0.00009298 | 3.0993e-05 | 2.8923 | 0.040094 * |
| **Prox** | 4.6 | 0.007175 ** | 0.0061884 | 0.0020628 | 1.8942 | 0.1413807 | 0.0032532 | 0.0010844 | 0.9958 | 0.4030339 | 0.0213231 | 0.0071077 | 6.5267 | 0.0008248 *** |
| **StatVic** | - | - | - | - | - | - | - | - | - | - | - | - | - | - |
| **Aggregate** | - | - | - | - | - | - | - | - | - | - | - | - | - | - |

**Supplementary *Table S6.*** *Post hoc test results of the linear mixed models. Multiple comparison of means with Tukey contrasts (adjusted p values with Holm-Bonferroni method).*

|  | | | | | | | |
| --- | --- | --- | --- | --- | --- | --- | --- |
| **Model** | **Fixed Factor** | | | **Estimate** | **Std. Erroer** | **Z value** | **p-value** |
| **Groom** | **Sex** | F > M | F > F | -0.009772 | 0.005516 | -1.771 | 0.152990 |
|  |  | M > F | F > F | -0.011541 | 0.005516 | -2.092 | 0.109257 |
|  |  | M > M | F > F | -0.021633 | 0.005718 | -3.783 | 0.000929 *** |
|  |  | M > F | F > M | -0.001770 | 0.004273 | -0.414 | 0.678722 |
|  |  | M > M | F > M | -0.011861 | 0.003836 | -3.092 | 0.009949 ** |
|  |  | M > M | M > F | -0.010092 | 0.003836 | -2.630 | 0.034107 * |
|  | **Origin** | captive > wild | captive > captive | -0.008543 | 0.004705 | -1.816 | 0.2776 |
|  |  | wild > captive | captive > captive | -0.011767 | 0.004705 | -2.501 | 0.0619 . |
|  |  | wild.wild | captive.captive | -0.014765 | 0.005085 | -2.904 | 0.0221 * |
|  |  | wild > captive | captive > wild | -0.003224 | 0.004385 | -0.735 | 0.9243 |
|  |  | wild > wild | captive > wild | -0.006221 | 0.004229 | -1.471 | 0.4239 |
|  |  | wild.wild | wild > captive | -0.002997 | 0.004229 | -0.709 | 0.9243 |
|  | **PHCinfant** | with > without | with > with | -0.007111 | 0.004846 | -1.467 | 0.7115 |
|  |  | without > with | with > with | -0.005075 | 0.004846 | -1.047 | 0.8851 |
|  |  | without > without | with > with | -0.010602 | 0.004267 | -2.485 | 0.0778 |
|  |  | without > with | with > without | 0.002036 | 0.004533 | 0.449 | 0.8851 |
|  |  | without > without | with > without | -0.003491 | 0.004281 | -0.816 | 0.8851 |
|  |  | without > without | without > with | -0.005528 | 0.004281 | -1.291 | 0.7866 |
| **Affil** | **Sex** | F > M | F > F | -0.0037370 | 0.0014428 | -2.590 | 0.0480 * |
|  |  | M > F | F > F | -0.0038567 | 0.0014428 | -2.673 | 0.0451 * |
|  |  | M > M | F > F | -0.0029556 | 0.0014956 | -1.976 | 0.1926 |
|  |  | M > F | F > M | -0.0001197 | 0.0011175 | -0.107 | 1.0000 |
|  |  | M > M | F > M | 0.0007814 | 0.0010035 | 0.779 | 1.0000 |
|  |  | M > M | M > F | 0.0009011 | 0.0010035 | 0.898 | 1.0000 |
|  | **Origin** | captive > wild | captive > captive | 0.0038393 | 0.0012307 | 3.120 | 0.00905 ** |
|  |  | wild > captive | captive > captive | 0.0039185 | 0.0012307 | 3.184 | 0.00871 ** |
|  |  | wild.wild | captive.captive | 0.0030425 | 0.0013300 | 2.288 | 0.08864 . |
|  |  | wild > captive | captive > wild | 0.0000792 | 0.0011469 | 0.069 | 1.00000 |
|  |  | wild > wild | captive > wild | -0.0007968 | 0.0011063 | -0.720 | 1.00000 |
|  |  | wild.wild | wild > captive | -0.0008760 | 0.0011063 | -0.792 | 1.00000 |
|  | **PHCinfant** | with > without | with > with | -0.0054868 | 0.0012676 | -4.328 | 7.51e-05 *** |
|  |  | without > with | with > with | -0.0053150 | 0.0012676 | -4.193 | 0.00011 *** |
|  |  | without > without | with > with | -0.0066599 | 0.0011161 | -5.967 | 1.45e-08 *** |
|  |  | without > with | with > without | 0.0001718 | 0.0011855 | 0.145 | 0.88479 |
|  |  | without > without | with > without | -0.0011731 | 0.0011197 | -1.048 | 0.68915 |
|  |  | without > without | without > with | -0.0013449 | 0.0011197 | -1.201 | 0.68915 |
| **Prox** | **Sex** | F > M | F > F | -0.0585887 | 0.0146360 | -4.003 | 0.000313 *** |
|  |  | M > F | F > F | -0.0588200 | 0.0160873 | -3.656 | 0.001023 ** |
|  |  | M > M | F > F | -0.0686524 | 0.0165764 | -4.142 | 0.000207 *** |
|  |  | M > F | F > M | -0.0002313 | 0.0131409 | -0.018 | 1.00000 |
|  |  | M > M | F > M | -0.0100637 | 0.0121463 | -0.829 | 1.00000 |
|  |  | M > M | M > F | -0.0098324 | 0.0101732 | -0.967 | 1.00000 |

Signif. codes: 0 ‘***’ 0.001 ‘**’ 0.01 ‘*’ 0.05 ‘.’ 0.1 ‘ ’ 1

**Supplementary Figure S1.** Plot of the residual normality distribution.


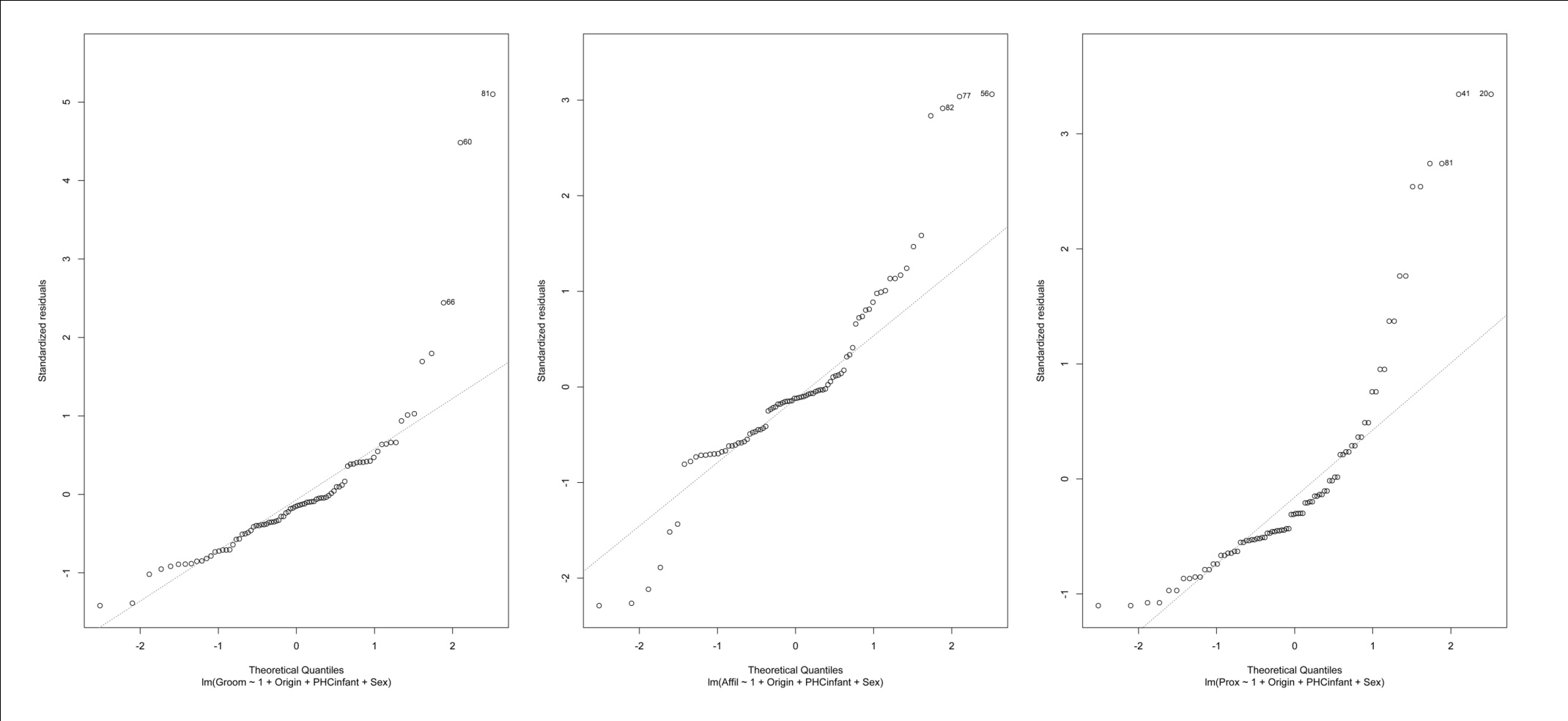


**Supplementary Figure S2.** Plot of the residual vs. fitted values.


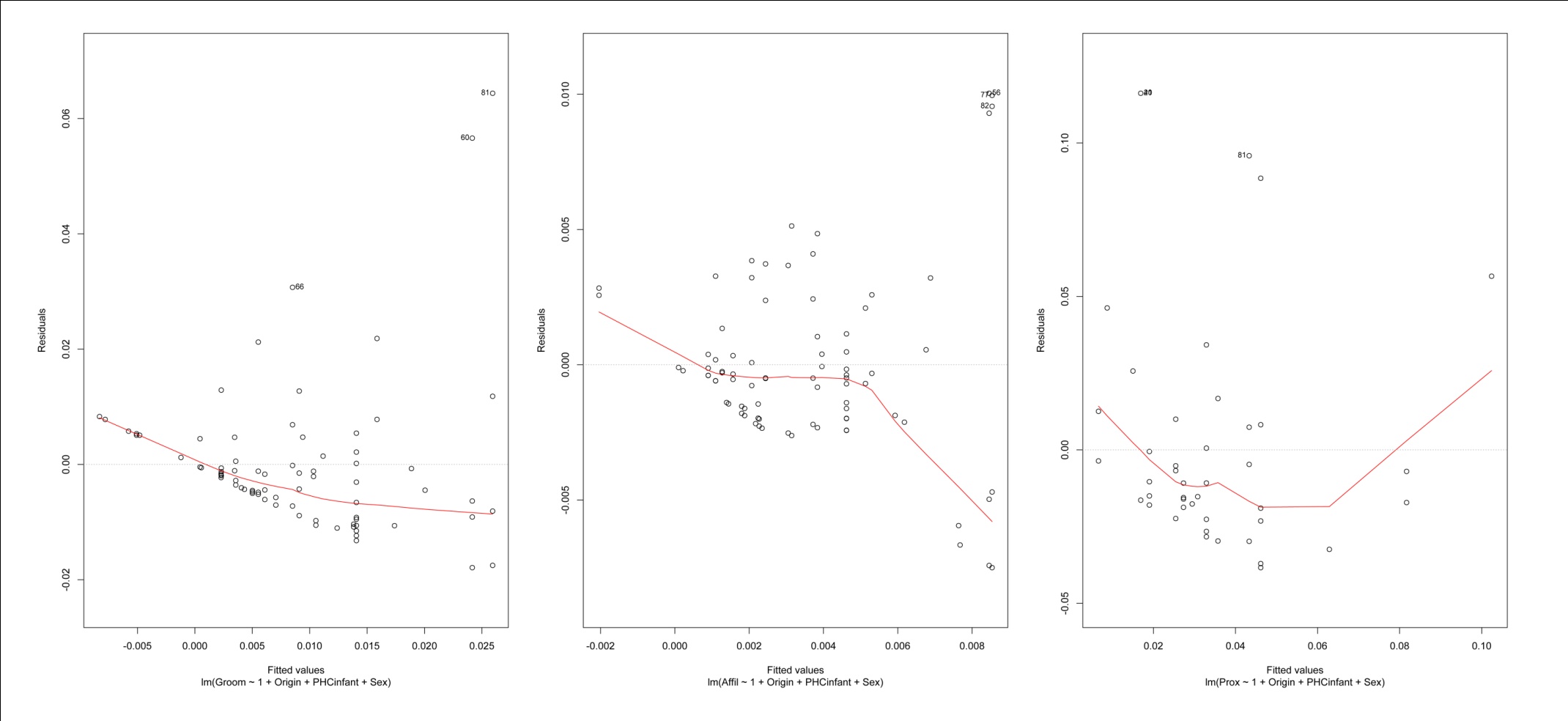


**References**

Domenico M, Solé-Ribalta A, Omodei E, Gómez S, Arenas A (2015) Ranking in interconnected multilayer networks reveals versatile nodes. Nature Communications 6:6868 doi:10.1038/ncomms7868
